# Cell-Type Specific Responses to DNA Replication Stress in Early *C*. *elegans* Embryos

**DOI:** 10.1371/journal.pone.0164601

**Published:** 2016-10-11

**Authors:** Holly Stevens, Ashley B. Williams, W. Matthew Michael

**Affiliations:** Molecular and Computational Biology Section, Department of Biological Sciences, University of Southern California, Los Angeles, CA 90089, United States of America; University of Minnesota Twin Cities, UNITED STATES

## Abstract

To better understand how the cellular response to DNA replication stress is regulated during embryonic development, we and others have established the early *C*. *elegans* embryo as a model system to study this important problem. As is the case in most eukaryotic cell types, the replication stress response is controlled by the ATR kinase in early worm embryos. In this report we use RNAi to systematically characterize ATR pathway components for roles in promoting cell cycle delay during a replication stress response, and we find that these genetic requirements vary, depending on the source of stress. We also examine how individual cell types within the embryo respond to replication stress, and we find that the strength of the response, as defined by duration of cell cycle delay, varies dramatically within blastomeres of the early embryo. Our studies shed light on how the replication stress response is managed in the context of embryonic development and show that this pathway is subject to developmental regulation.

## Introduction

Chromosome replication during S-phase of the cell cycle is a tightly regulated process that must occur in an error-free manner if genome stability is to be maintained during cell division. Cells can encounter replication stress when the progress of DNA polymerases on the template strand is hindered by such roadblocks as DNA damage, tightly bound proteins, nucleotide exhaustion, tri-nucleotide repeats, or collisions with transcribing RNA polymerases [1]. The ability of cells to properly manage replication stress is critical to their ability to faithfully transmit their genetic material to daughter cells at mitosis. To maintain genome stability, cells activate a so-called checkpoint response to replication stress, and this results in delayed cell cycle progression as well as stabilization of stalled replication forks [2–5]. Much of what is known about the replication stress response has come from studies on homogenous populations of yeast or mammalian tissue culture cells, and comparatively little is known about the replication stress response in an organismal context. Here, we seek to further establish the *C*. *elegans* early embryo as a model system with which to study the replication stress response in the context of a developing organism.

The ATR protein kinase (ATL-1 in *C*. *elegans*) is the master regulator of the replication stress response [2–5]. Its activation at sites of replication stress requires multiple factors, including ssDNA that is coated with the trimeric RPA protein, the 911 clamp protein (HPR-9, MRT-2, and HUS-1 in *C*. *elegans*), the clamp loader RAD17 (HPR-17 in *C*. *elegans*), and the TOPBP1 protein (MUS-101 in *C*. *elegans*) [2–5]. Upon activation, ATR requires the function of mediator proteins, such as CLASPIN and the TIMELESS-TIPIN complex, to access its substrates [6–8]. One critical ATR substrate is the CHK1 (CHK-1 in *C*. *elegans*) protein kinase. ATR-mediated phosphorylation of CHK1 activates CHK1 kinase activity and thereby allows the protein to delay cell cycle progression [2–5]. In addition to these highly conserved components, in *C*. *elegans*, the WRN-1 helicase also plays an important role in the ATR pathway [9–11]. WRN-1 is the worm ortholog of the human Werner syndrome helicase, and it has been shown to act upstream of ATR during the replication stress response, where it is required for docking of ATL-1/ATR to sites of replication stress [10–11]. One possibility, given its role in ATL-1/ATR docking, is that WRN-1 assumes a role similar to the ATR binding partner ATRIP. A *C*.*elegans* ortholog for ATRIP has not yet been identified.

Previous works have examined the replication stress response in early *C*. *elegans* embryos. These studies showed that treatment of embryos with the replication stress inducer hydroxyurea (HU) delays progression through the cell cycle in an ATL-1 and CHK-1 dependent manner [12]. Similar findings have been reported for strains harboring mutations in components of the replication machinery, for example a regulatory subunit for DNA polymerase alpha (*div-1*) [13] or the *sld-2* initiation factor [14]. Furthermore, previous studies showed that, in two-cell embryos, the germline progenitor P1 cell was more delayed than was its sister, the somatic precursor AB cell [12–13]. These data suggest that the ATR pathway is somehow more active in P1 relative to AB, however the basis for this is not known. In this study, we extend these previous works in two directions. First, we examine the requirement for multiple ATR pathway components in replication stress-induced cell cycle delay, so that a fuller picture of how this pathway functions in the embryo could be achieved. Second, in order to learn more about how the replication response is regulated developmentally, we examine how seven distinct embryonic blastomeres respond to stress, and results show a surprising degree of variance in these responses. Taken together, these two lines of analysis combine to further establish the early worm embryo as an important model for how the replication stress response functions in a developmental context.

## Results and Discussion

### Measuring the replication stress response in *C*. *elegans* zygotes

**Fig 1:**
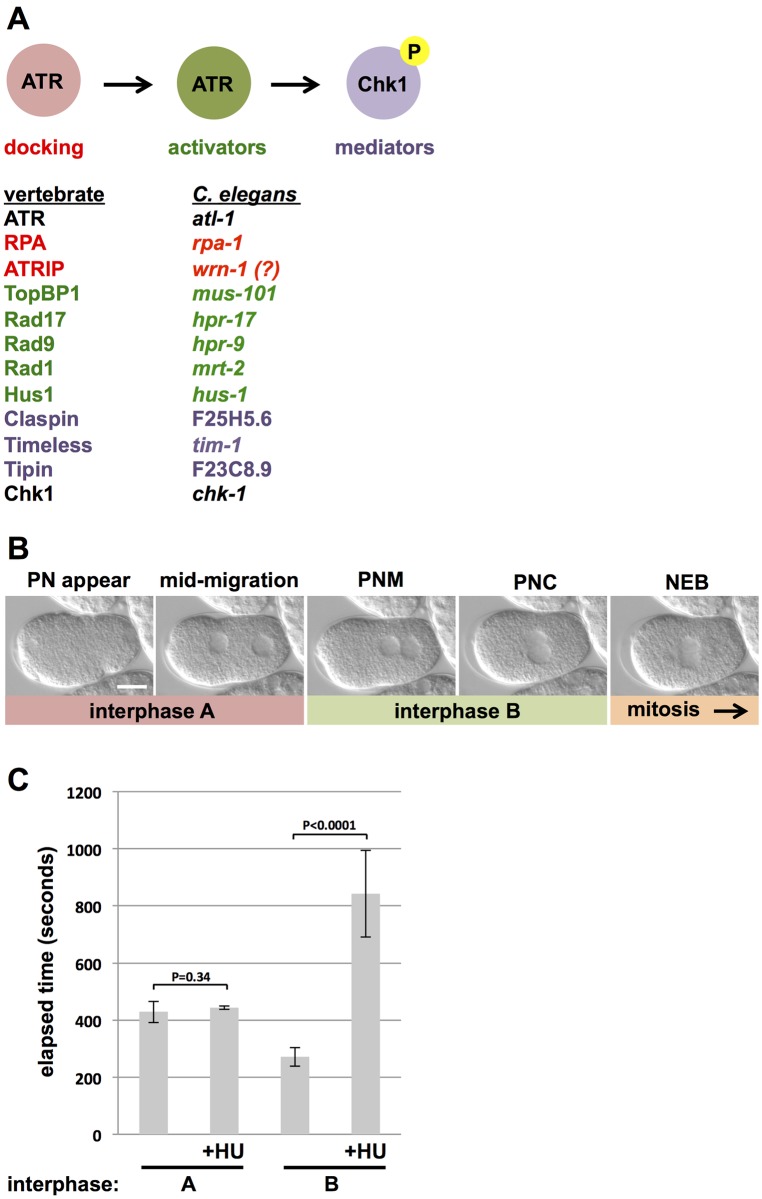
ATR pathway overview in *C*. *elegans* and demonstration that replication stress does not delay pronuclear migration in one-cell embryos. (A) Overview of ATR pathway. Components of the ATR pathway are divided into the functional categories “docking”, “activators”, and “mediators”. In addition, components of the ATR pathway are listed along with the corresponding ortholog in *C*. *elegans*. (B) DIC images of a zygote progressing through the first cell cycle are shown. Distinct stages of this process are named based on the position of the pronuclei. “PN appear” refers to the first appearance of pronuclei, when imaged using DIC optics. “Mid-migration” refers to the stage where the maternal pronucleus has progressed halfway across the embryo length. “PNM” refers to pronuclear meeting. “PNC” refers to pronuclear centration. “NEB” refers to nuclear envelope breakdown. As detailed in the Results and Discussion section, we arbitrarily divided interphase of the first cell cycle into “A” and “B” portions; NEB marks the onset of mitosis. Anterior is to the left in all images. Bar = 10 uM. (C) The interphase A and B portions of the first cell cycle were timed after optional exposure to HU. For each data point, ten embryos were timed over two independent biological replicates (5/replicate). For this and all subsequent figures, error bars refer to one standard deviation from the mean and P values determined by Student’s *t*-test.

In a first line of investigation, we used RNAi to knockdown several different orthologs of the core ATR pathway components (Fig 1A) and then challenged these embryos with replication stress followed by timing of the first mitotic cell cycle. It was important to measure the replication stress response in the first mitotic cycle, as opposed to later cycles, because examining later cycles is complicated by the fact that problems arising during the first cycle could carry over to subsequent cycles and this renders interpretation difficult. In *C*. *elegans*, newly fertilized embryos rapidly complete maternal meiosis and then both the maternal and paternal pronuclei immediately enter S-phase [15–16]. After DNA replication has finished the nuclei enter a G2-like phase. At this time the maternal pronucleus migrates across the embryo length and ultimately bumps into the posteriorly located paternal pronucleus (see panels labeled “mid-migration” and “PNM” for pronuclear meeting in Fig 1B). After PNM, the apposed pronuclei move towards the center of the embryo (pronuclear centration or PNC in Fig 1B), and shortly thereafter nuclear envelope breakdown occurs and the cell enters mitosis (NEB in Fig 1B).

We used differential interference contrast (DIC) microscopy to image early embryos. This technique allows high resolution imaging of key cellular events during the first cell cycle, but also renders timing of the complete first cycle challenging, as previously described [12], because the nuclei are not visible immediately after fertilization and the completion of meiosis. One solution to this problem has been to time a partial first cycle, using pronuclear position as landmarks for cell cycle progression. For example, previous work has timed the interval between PNM and NEB [17], however this approach is only applicable here if replication stress does not impact the timing of maternal pronuclear migration. To determine this, we arbitrarily divided interphase of the first cell cycle into two portions. Interphase A corresponds to the time between the first appearance of pronuclei (panel “PN appear” in Fig 1B) and PNM, whereas interphase B corresponds to the time between PNM and NEB (Fig 1B). If the timing of pronuclear migration is blind to replication stress, then we expect the stress-inducer HU to increase the time required for interphase B, but not interphase A. Animals were optionally treated with HU, as described in the Materials and Methods, and the times required for interphases A and B were recorded for 10 control samples and 10 HU-treated samples. As shown in Fig 1C, HU did not affect the timing of interphase A (ie the time required for the maternal pronucleus to migrate across the embryo length), but did significantly delay the time between PNM and NEB. This experiment shows that maternal pronuclear migration can be uncoupled from progression through S-phase, and therefore that the effect of replication stress on cell cycle timing can be reliably assessed by measuring the time interval between mid-migration and NEB.

**Fig 2:**
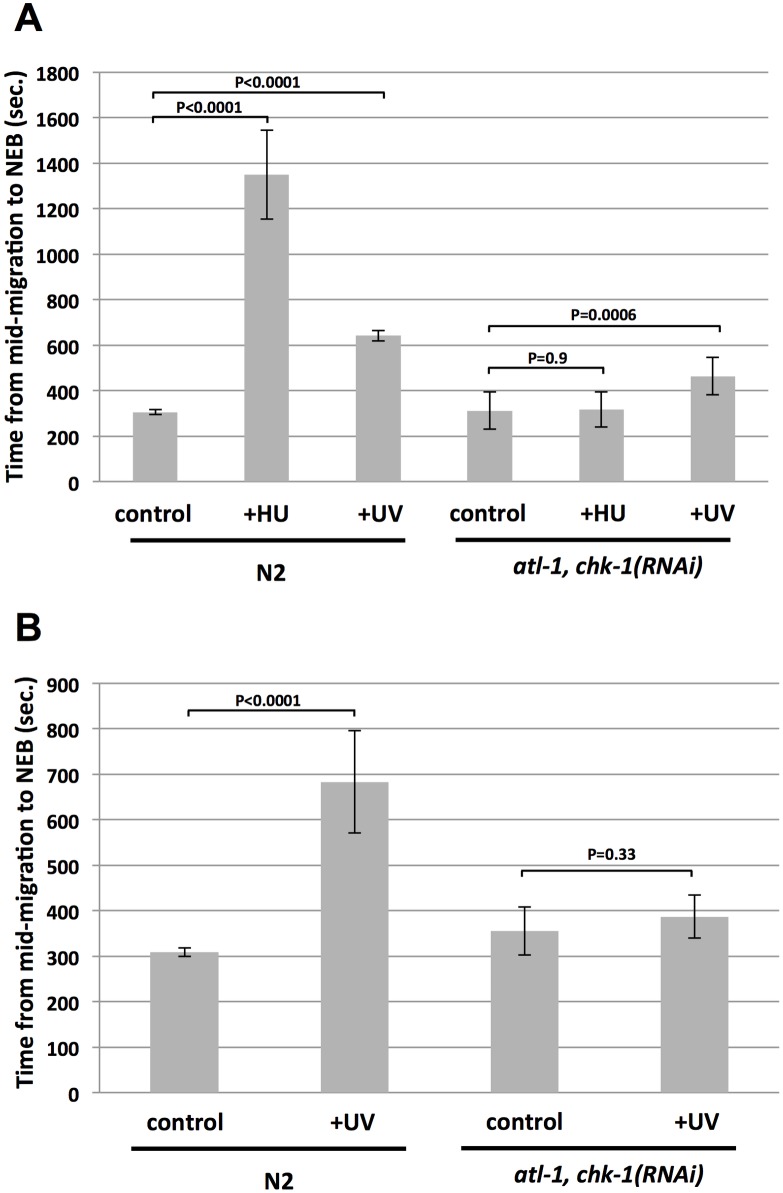
The ATR pathway controls cell cycle delay after HU or UV-C mediated replication stress in one-cell embryos. (A) Either wild type (N2) or *atl-1*,*chk-1(RNAi)* embryos were optionally treated with either HU or UV-C, as indicated. The time required for embryos to progress from the mid-migration to the NEB stage was then recorded. For each data point, ten embryos were timed over two independent biological replicates (5/replicate) and the values were then averaged and plotted. (B) Same as (A) except HU treatment was omitted and 5 embryos were timed per data point over one biological replicate.

One goal of this study was to examine the requirements for presumed ATR pathway components in replication stress-induced cell cycle delay in early embryos, using RNAi to deplete the component of interest. We first examined the impact of RNAi-mediated co-depletion of *atl-1* (ATR) and *chk-1* (CHK1). Previous work has shown that co-depletion of both *atl-1* and *chk-1* is the most effective way of attenuating the ATR-CHK1 axis in the early embryo, relative to either single depletion alone [12]. Replication stress was induced either by HU treatment or UV-C (254 nM) light. Previous work from our group has shown that a large dose of UV-C is required to delay the embryonic cell cycle in *C*. *elegans*, owing to the unusually high capacity these embryos have for trans-lesion synthesis, which directly replicates damaged DNA and thereby prevents replication forks from stalling [18,19]. Accordingly, we irradiated the samples with 112 J/m^2^ of UV-C. As shown in Fig 2A, both HU and UV-C delayed the first cell cycle in wild type embryos. In the absence of stress, embryos required ~300 seconds to progress from mid-migration to NEB, and this was increased by HU to nearly 1400 seconds and by UV-C to ~600 seconds. By contrast, for *atl-1*,*chk-1(RNAi)* embryos, HU did not significantly extend the cell cycle while UV-C did, but only modestly. The data in Fig 2A suggest that some component of cell cycle delay after UV-C might occur independently of the ATR pathway, given that the UV-C delay was not absolutely reduced by *atl-1*,*chk-1* RNAi. This is not the case, however, as in an independent experiment we observed that *atl-1*,*chk-1* RNAi could indeed significantly reverse a UV-C induced delay (Fig 2B). These data show that the ATR pathway controls cell cycle delay after either HU- or UV-C induced replication stress. We note that previous work had shown a requirement for *atl-1* and *chk-1* in cell cycle delay after HU [12], however the requirement for *atl-1* and *chk-1* in cell cycle delay after UV-C had not been previously reported. We also examined timing of the first cell cycle in wild type animals and compared them to those experiencing “vector-only” RNAi, whereby animals are fed bacteria containing the dsRNA expression vector alone. We found that the two samples were indistinguishable across all conditions tested (S1 Fig), showing that the RNAi procedure alone does not effect cell cycle timing or the replication stress response.

### Analysis of ATR docking factors

**Fig 3:**
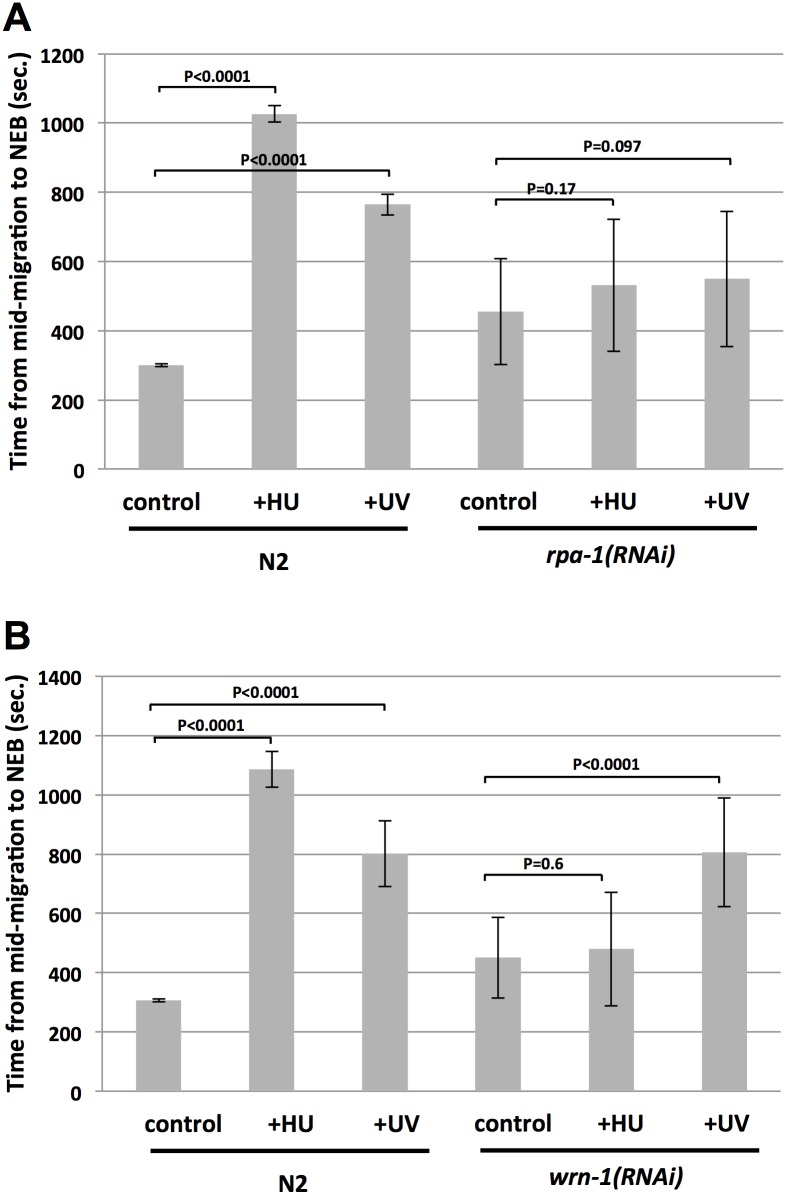
Analysis of RPA-1 and WRN-1 for cell cycle delay after replication stress. (A) Either wild type (N2) or *rpa-1(RNAi)* embryos were optionally treated with either HU or UV-C, as indicated. The time required for progression from the mid-migration to the NEB stage was then recorded. For each data point, twenty embryos were timed over two independent biological replicates (10/replicate) and the values were then averaged and plotted. (B) Same as (A) except *wrn-1(RNAi)* embryos were examined.

We next analyzed different components of the ATR pathway in *C*. *elegans* for roles in HU- or UV-C induced cell cycle delay in one-cell embryos. We first grouped these components into functional categories, based on whether they are thought to function in ATR docking to sites of replication stress, or in ATR activation, or in mediating the ability of active ATR to access its substrates (Fig 1A), and we then tested two genes from each category. The *rpa-1* gene encodes the *C*. *elegans* ortholog of the large, 70 kDa subunit of the ssDNA-binding protein RPA. Previous work in *C*. *elegans* has shown that RPA-1 is required for recruitment of ATL-1 to sites of replication stress [20], and that it physically interacts with WRN-1 [11]. A role for RPA in ATR recruitment is evolutionarily conserved from yeast to man [21]. As shown in Fig 3A, *rpa-1(RNAi)* embryos were modestly slower than their wild type counterparts in the absence of replication stress. Importantly, after replication stress, wild type embryos showed typical patterns of cell cycle delay, whereas *rpa-1(RNAi)* embryos were not significantly delayed, by either HU or UV-C (Fig 3A). These data suggest that RPA-1 is required for ATR pathway function in the early embryo.

We next examined WRN-1 function during the replication stress response in one-cell embryos. Previous work has shown that WRN-1 depletion accelerates the embryonic cell cycle at the two-cell stage [9], and that the protein is required for CHK-1 phosphorylation in adult germ cells after HU, but not after UV light [10]. We observed that *wrn-1(RNAi)* embryos actually showed a slight delay in the first cell cycle, relative to wild type (Fig 3B). We did, however, observe accelerated cell cycles at the two-cell stage (S2 Fig), which is consistent with previous reports [9]. When replication stress was applied, we found that *wrn-1* depletion prevented an HU-imposed cell cycle delay, however UV-C could still impose a delay (Fig 3B). We conclude that WRN-1 functions within the ATR pathway when replication is stressed by nucleotide deprivation, but not when DNA polymerases are delayed by UV-C imposed lesions. This finding suggests that the ATR pathway in the early embryo differs depending on the type of replication stress the embryo experiences, a point that is borne out by additional findings, detailed below. We note that the difference in response to HU relative to UV observed for *wrn-1(RNAi)* embryos could be due to the RNAi working less efficiently in the embryos exposed to UV relative to those exposed to HU, however we consider this unlikely given that the same batch of RNAi embryos was used for both treatments. It is also possible that more WRN-1 protein is needed for the UV response relative to the HU response.

### Analysis of ATR activators

**Fig 4:**
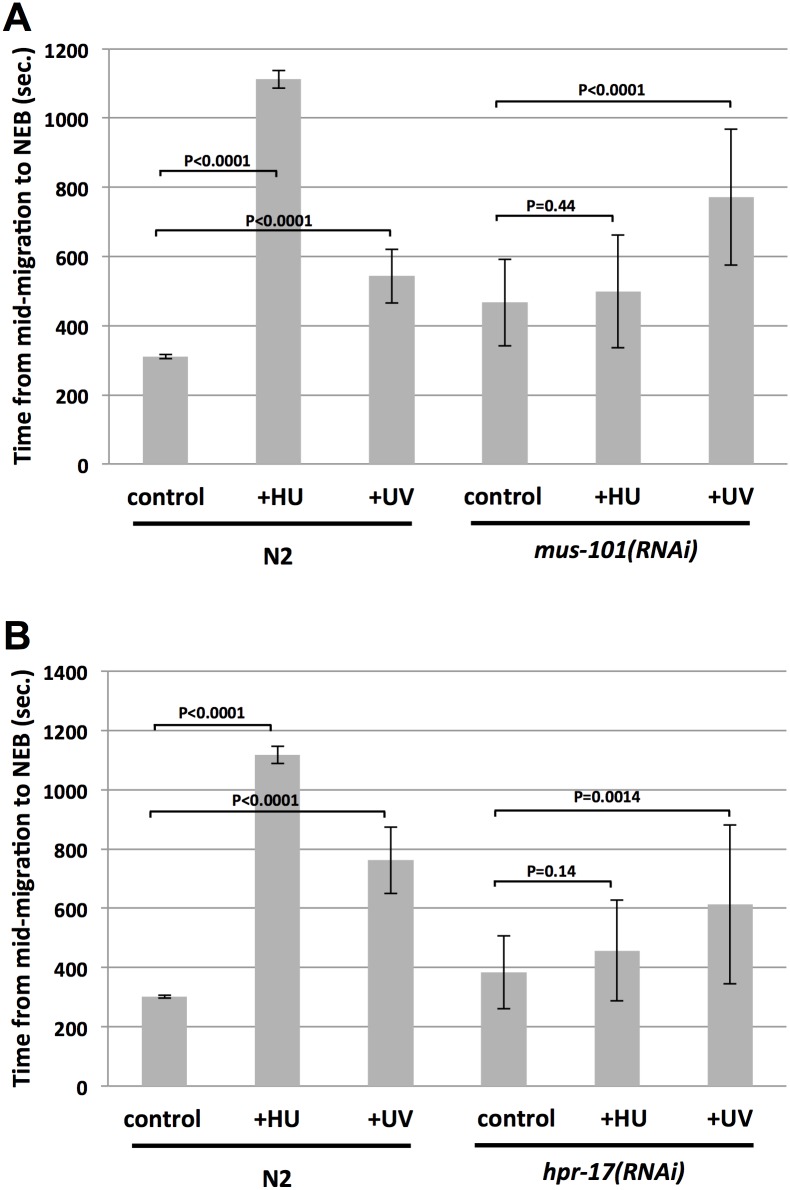
Analysis of MUS-101/TOPBP1 and HPR-17/RAD17 for cell cycle delay after replication stress. (A) Either wild type (N2) or *mus-101(RNAi)* embryos were optionally treated with either HU or UV-C, as indicated. The time required for embryos to progress from the mid-migration to the NEB stage was then recorded. For each data point, twenty embryos were timed over two independent biological replicates (10/replicate) and the values were then averaged and plotted. (B) Same as (A) except *hpr-17(RNAi)* embryos were examined.

ATR activators were examined next, and these included MUS-101 (TOPBP1) and HPR-17 (RAD17). Previous work from our group has shown that MUS-101 is required for DNA replication in the early embryo [22]. TOPBP1 orthologs in other organisms all play dual roles in DNA replication and checkpoint activation [23], and thus we considered it likely that MUS-101 would be needed for ATR pathway function in *C*. *elegans*, however this had never been tested. Compared to control samples, *mus-101(RNAi)* embryos displayed a slower first cell cycle (Fig 4A). When challenged with HU, control embryos showed a robust delay, however a delay was not observed in *mus-101(RNAi)* samples. Interestingly, UV-C induced a delay in control samples and also did so in *mus-101(RNAi)* embryos. The UV-C induced delay for the *mus-101(RNAi)* embryos was deemed significant by the Student’s *t*-test (*P*<0.001). A very similar pattern was observed for *hpr-17(RNAi)* embryos, which were slower in general than controls and delayed by UV-C but not HU (Fig 4B). For these embryos, the UV-C induced delay was also deemed significant (*P* = 0.0014; Student’s *t*-test). Thus, for three ATR pathway components, WRN-1, MUS-101, and HPR-17, a role could be uncovered for HU-induced cell cycle delay but not UV-C induced delay. However, we did observe a requirement for ATL-1/CHK-1 in UV-C induced cell cycle delay (Fig 2B), and thus we conclude that in early *C*. *elegans* embryos ATR uses a distinct set of components for responses to HU relative to UV-C.

### Analysis of ATR mediators

**Fig 5:**
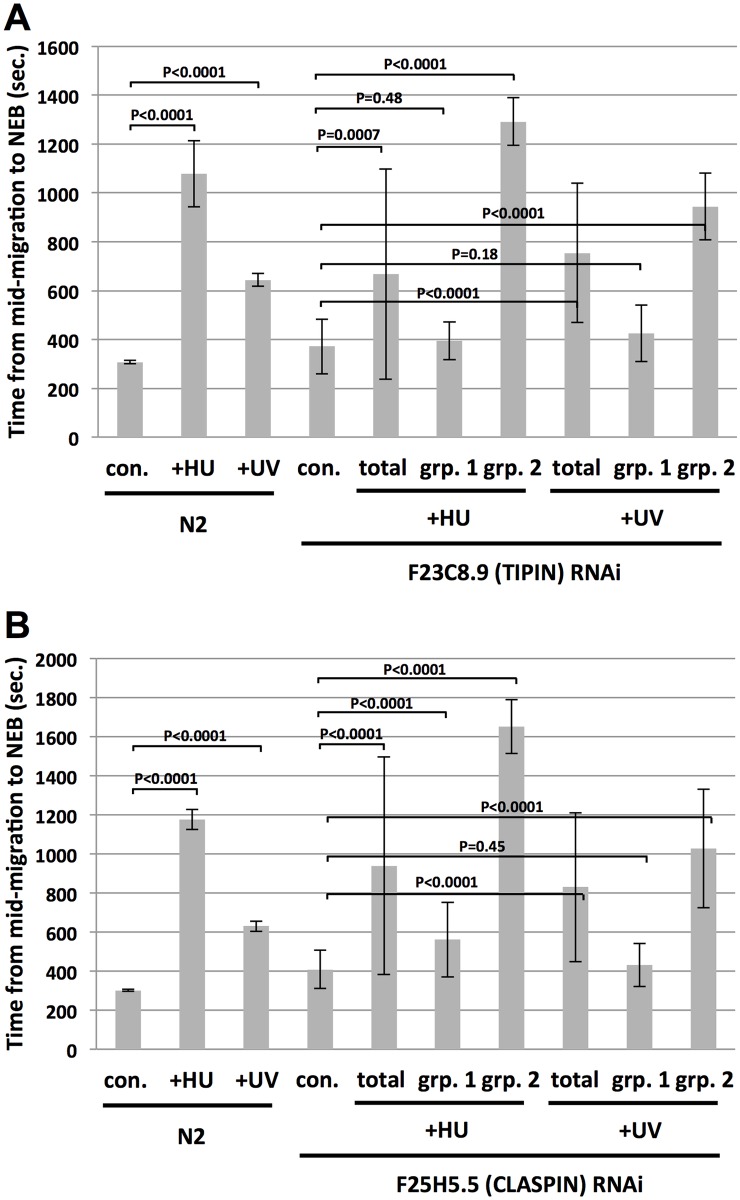
Analysis of F23C8.9/TIPIN and F25H5.5/CLASPIN for cell cycle delay after replication stress. (A) Either wild type (N2) or F23C8.9 RNAi embryos were optionally treated with either HU or UV-C, as indicated. The time required for embryos to progress from the mid-migration to the NEB stage was then recorded. For the N2 data points, 10 embryos were timed over two independent biological replicates (5/replicate) and the values were then averaged and plotted. For the F23C8.9 control samples (labeled “con.” for “control” in the graph), 20 samples were averaged from timings taken over two biological replicates (10/replicate). For HU-treated samples, the data point labeled “total” reflects the average of 23 recordings, “grp. 1” represents the 16 fastest samples, and “grp.2” the 7 slowest samples. For UV-C treated samples, the data point labeled “total” reflects the average of 30 recordings, “grp. 1” represents the 11 fastest samples, and “grp.2” the 19 slowest samples. (B) Same as (A) except F25H5.5 RNAi embryos were examined. For the N2 data points, 10 embryos were timed over two independent biological replicates (5/replicate) and the values were then averaged and plotted. For the F25H5.5 RNAi control samples (labeled “con.” for “control” in the graph), 20 samples were averaged from timings taken over two biological replicates (10/replicate). For HU-treated samples, the data point labeled “total” reflects the average of 39 recordings, “grp. 1” represents the 25 fastest samples, and “grp.2” the 14 slowest samples. For UV-C treated samples, the data point labeled “total” reflects the average of 45 recordings, “grp. 1” represents the 15 fastest samples, and “grp.2” the 30 slowest samples.

The *C*. *elegans* genome contains clear orthologs for two different ATR mediators, Claspin and the Timeless/Tipin complex. Timeless/Tipin has multiple cellular functions, including chromosome cohesion, circadian clock control, replication fork protection, and mediating ATR kinase activity [7,8]. Previous work has shown that the Tipin component of the human complex is dispensable for chromosome cohesion [24], and thus we targeted this factor in our analysis of early embryo checkpoint control. *C*. *elegans* Tipin is encoded by the previously uncharacterized F23C8.9 locus. RNAi against F23C8.9/Tipin did not alter an unperturbed cell cycle in one-cell embryos (Fig 5A), however a complex pattern emerged when these embryos were challenged with replication stress. For HU treatment, we observed a high degree of variance in the timing data, as indicated by the very large error bars on the graph in Fig 5A (sample labeled “total”). Inspection of this data set revealed two clear groups of timings, one group was very fast (grp. 1 in Fig 5A) and the other very slow (grp. 2 in Fig 5A), and the variance within these groups was dramatically reduced relative to the total. Similar results were obtained after challenge with UV-C (Fig 5A). While it is difficult to draw hard and fast conclusions from this complex data set, it does seem likely that F23C8.9/Tipin plays a role in cell cycle delay after both HU and UV-C, as evidenced by the groups of fast embryos. The groups of slow embryos are potentially explained by a role for F23C8.9/Tipin in replication fork protection, as detailed below. Interestingly, a very similar pattern was observed for RNAi against the Claspin ortholog F25H5.5, where replication stress caused a high degree of variance within the timings, and these timings clearly segregated into fast and slow groupings (Fig 5B). It thus seems likely that F25H5.5/Claspin also plays a role in both HU- and UV-C induced cell cycle delay.

How might a single RNAi treatment produce such very different phenotypes (fast versus slow embryos after replication stress) for both F23C8.8/Tipin and F25H5.5/Claspin? Importantly, both proteins play dual roles in ATR signaling and replication fork protection [6–8]. If different protein levels are needed for these two functions, for example perhaps less protein is required for ATR signaling relative to fork protection, then the RNAi could vary in its ability to compromise both functions, relative to fork protection alone. In other words, the very fast group of embryos can be explained by an RNAi efficacy that eliminates both ATR signaling and fork protection functions, whereas the very slow group is explained by an RNAi efficacy that eliminates the fork protection function but not the ATR signaling function. In this latter scenario, replication stress stalls the forks, and their protection is compromised but ATR signaling is intact and thus the delay is even longer than that observed in control samples. Clearly, further work is needed to unravel the roles of F23C8.8/Tipin and F25H5.5/Claspin in the early embryo, however the data presented here do strongly suggest a role for both proteins in cell cycle delay after both HU and UV-C.

### Cell type specific responses to replication stress in the early embryo

In a second line of investigation we wanted to determine if the replication stress response is developmentally regulated in the early embryo. This has been suggested by previous studies looking at two-cell embryos, where the response in the anterior AB cell differs from that of its sister cell, P1 [12,13, 25]. Whether this represents a true developmental difference between the AB and P1 cells, or conversely is due to problems that occurred during the first cell cycle being differentially inherited or managed by AB relative to P1, is not known. Furthermore, it is not known if differences in the replication stress response extend beyond the two-cell stage.

**Fig 6:**
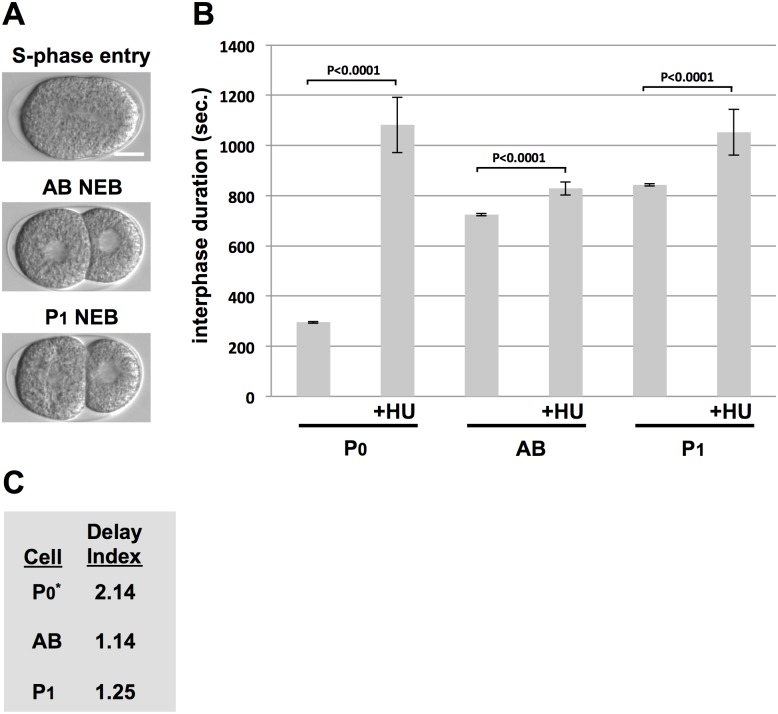
Comparing the HU response between the P0, AB, and P1 blastomeres. (A) Images depicting when cell cycle timing was started (top panel) and when it was terminated for AB (middle panel) or P1 (bottom panel). Bar = 10 uM. (B) N2 animals were optionally treated with HU and interphase duration for a given cell type was plotted. Each data point represents the average of 10 embryos obtained over two biological replicates (5/replicate). (C) The Delay Indices (DI) for the given cell types are depicted. Please see the main text for how the DIs were calculated.

To begin this analysis, we first examined the response to HU in one- and two-cell embryos. To time AB and P1 we used the standard method, whereby S-phase entry is visualized in the DIC microscope by cortical ingression [25]. This membrane invagination occurs during cytokinesis of the zygote and is known to coincide with the onset of DNA synthesis in AB and P1 [15]. Timing was initiated at cortical ingression and continued until NEB in AB and P1, respectively (Fig 6A). Using our standard HU treatment protocol (see Materials and Methods), we timed a portion of the P0 cell cycle, from mid-migration to NEB, as well as the complete duration of interphase in AB and P1 (Fig 6B). As expected, HU extended the cell cycle in all three cases, but the degree to which extension occurred varied in an interesting way. We developed the “delay index” (DI) as a means to quantify the effect a given replication stress inducer has on a given cell type. The DI is simply the time of cell cycle duration in the stressed condition over the duration without stress, and thus the higher the DI value the more a given cell is delayed by the stress. Calculating the DIs for AB and P1 is simple; for P0, however, we could only generate approximate values, as detailed in the Materials and Methods, and thus an asterisk appears next to P0 in Fig 6C.

We see that in two-cell embryos, the P1 DI is larger than the AB DI, as expected given previous work (Fig 6C) [12,13,25]. We also see that the P0 DI is larger than that of P1, suggesting that the checkpoint response to nucleotide deprivation is weakened in both of P0’s daughter cells. One explanation for this is a dilution of ATR pathway components by cell division that would reduce their concentration in AB and P1 relative to the P0 mother. Alternatively, the replication stress response may be actively tuned down in the AB and P1 daughters, a possibility that is supported by data presented below. Interestingly, P0 is the only early cycle that contains a gap phase—it has a G2 that is roughly the same length as S-phase [16]. The G2 phase is deleted after the first cell cycle, and it is interesting to speculate that the loss of G2 and a weakened checkpoint response in the P0 daughter cells are mechanistically related.

**Fig 7:**
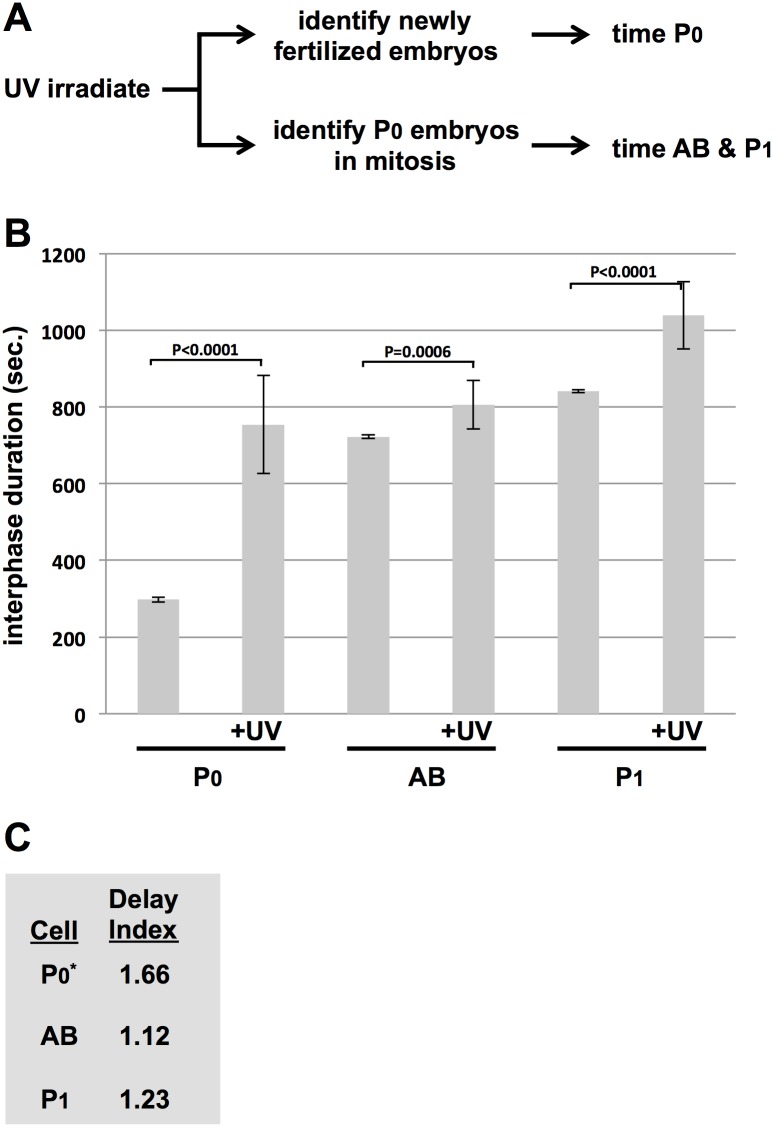
Comparing the UV-C response between the P0, AB, and P1 blastomeres. (A) Schematic depicting the experimental strategy, whereby UV-C induced replication stress is applied just prior to the S-phase of interest. (B) N2 animals were optionally treated with UV-C and interphase duration for a given cell type was plotted. Each data point represents the average of 10 embryos obtained over two biological replicates (5/replicate). (C) The Delay Indices (DI) for the given cell types are depicted.

One concern with using HU to induce stress is that events occurring in later cycles are likely to be influenced by events that happened in previous cycles, rendering analysis of the later cycles challenging. To get around this, we required a scheme whereby stress could be rapidly induced just prior to the S-phase of interest. For this, UV-C was the obvious choice, as the time required to induce the stress is on the order of seconds. To time P0, we irradiated the animals and then identified newly fertilized embryos for analysis (Fig 7A). For AB and P1, we irradiated animals and then immediately dissected out the embryos. P0 samples in mitosis were rapidly identified and these samples were timed upon completion of mitosis. This scheme thus allows the analysis of the very first S-phase after the induction of UV-C mediated DNA damage, in all three cell types. Timing was performed and analyzed as in Fig 6. As shown in Fig 7B & 7C, we again observed a hierarchy of responses, with P0 at the top (DI = 1.66), P1 in the middle (DI = 1.23), and AB, with the weakest response, at the bottom (DI = 1.12). As was the case with HU, the P1 response is closer to its sister AB than its mother P0, showing again that the replication stress response is remodeled after cell division in the early embryo. Taken together, our data show conclusively that the replication stress response does indeed vary between cell types in the early embryo, and is thus that the response is under developmental control.

**Fig 8:**
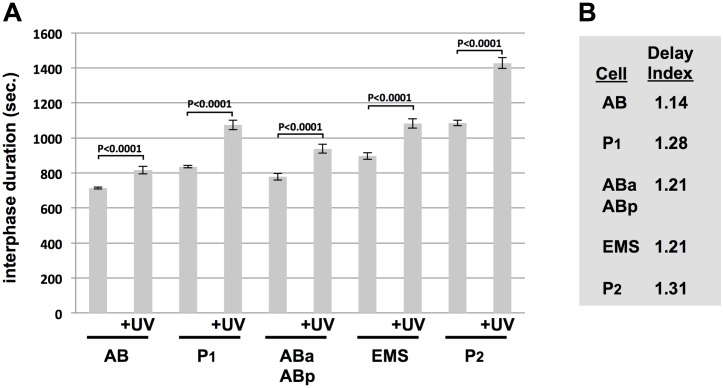
Comparing the UV-C response between the AB, P1, ABa, ABp, EMS, and P2 blastomeres. (A) Samples were optionally treated with UV-C and interphase duration for the indicated cell types is plotted. We note that ABa and ABp divide in a highly synchronous manner and thus their timings are grouped together. Each data point represents the average of 10 embryos obtained over two biological replicates (5/replicate). (B) The Delay Index (DI) for the given cell types is depicted.

In a final experiment we extended our developmental analysis of the replication stress response by examining an additional round of mitotic division. We used UV-C and timed interphase duration in AB and P1, and also in their division products, ABa, ABp, EMS, and P2. The results are shown in Fig 8A & 8B. Once again, the DI values varied in an interesting way and this allowed a couple of important principles to emerge. First, we see that the AB division products, ABa and ABp, displayed identical DIs, a value that is higher than that of their mother, AB. In addition, the P2 cell showed a higher DI than its mother, P1. These data show that DIs are not automatically decreased after cell division in the early embryo and this, in turn, rules out dilution of ATR pathway components after cell division as the mechanism by which DI values change after cell division. Second, we see that the germline progenitor P lineage always contains the highest DI value after a given round of cell division, showing that the strength of the replication stress response segregates with germline fate. This is a fascinating property of this system and one for which the mechanistic basis is not at all understood.

## Conclusions

In this work we identify two novel aspects of the replication stress response in early *C*. *elegans* embryos, and both findings open up exciting new pathways for discovery in the emerging field of how cell survival and stress response pathways are regulated during development. First, we have shown that the components of the ATR pathway differ dependent on the type of replication stress the embryo experiences. The ATR pathway components WRN-1, MUS-101, and HPR-17 are all required for cell cycle delay after HU but none are required for the response to UV-C. This suggests that the ATR pathway is activated by UV-C in a manner that is independent of the activator MUS-101/TOPBP1 and the 911 clamp loader HPR-17/RAD17. And by extension, it would appear that the 911 clamp itself is dispensable, give that its loader HPR-17 is dispensable. This, in turn, suggests a mode of ATR activation that is very different from that which has been described in human cells, where TOPBP1 and RAD17/911 play central roles in activation of ATR kinase under a wide variety of genotoxic stress conditions [2–5]. Future work in this area will focus on the means by which UV-C activates ATL-1/ATR in *C*. *elegans* embryos.

Another new path for discovery centers on how the replication stress response is regulated during early development in the worm. Previous works had noted a difference in the response at the two-cell stage, however whether this was due to a carry-over effects of damage suffered at the one-cell stage was never addressed. Here, we were able to show this point conclusively by utilizing UV-C to induce stress in a manner where the first cell cycle is allowed to occur in an unhindered manner and replication stress is thereby experienced for the first time at the two-cell stage. Under these conditions, we do indeed observe a difference between blastomeres at the two-cell stage (Fig 7). We go on to extend this analysis to the four-cell stage and here the results were illuminating as we see that the replication stress response is being constantly remodeled after each round of cell division. Using the duration of cell cycle delay as an indirect measure for the strength of the replication stress response, and DI values to reflect the duration of the delay, we see that P0’s daughter cells have a weaker response than their mother, whereas AB’s daughter cells have a stronger response than their mother. We also observe that the response is always strongest in P-cells within a given round of division. How the replication stress response is remodeled and the means by which a strong response segregates with germline fate are important questions for future research.

## Materials and Methods

### *C*. *elegans* strains and RNAi

The N2 strain [26] was used as wild type in all experiments. All RNAi was done by feeding [27]. All feeding vectors for RNAi experiments were obtained from the Ahringer RNAi Library (Source BioScience). To prepare the RNAi plates, standard nematode growth media plates were supplemented with 0.5 mM IPTG and 100 ug/ml carbenicillin. Plates were seeded with overnight cultures of HT115(DE3) transformed with the appropriate feeding vector and allowed to dry overnight. Plates were used the following day. For RNAi treatment, synchronized cultures of starved L1s, obtained by bleaching gravid adults followed by overnight incubation in M9 media, were seeded onto RNAi plates and then incubated for 60 hours at room temperature prior to analysis.

### Timing interphase duration in early embryos

Gravid adults were placed in a 5-ul drop of M9 on an 18 mm-square coverslip and sliced open with a 20-gauge needle to release embryos. Coverslips were then mounted on microscope slides containing a thin 1.8% agarose pad and embryos were imaged using an Olympus BX51 microscope equipped with DIC optics. Images shown in Figs 1B and 6A were captured using the 40X objective and a Spot camera (Diagnostic Instruments, Inc.). To calculate DIs for the P0 cell cycle, we needed to account for the portion of the cell cycle that was not directly measured in a given experiment, that being the time interval between pronuclear appearance and mid-migration. To do so, we timed this interval in 25 N2 embryos and obtained a value of 392.6 +/- 8.4 seconds. We then took this figure, 392.6 seconds, and added it to the experimentally determined values for P0 duration shown in Figs 6B & 7B. This gave an estimate of total cell cycle length, which was then used to calculate the P0 DIs. Because the time required for pronuclear migration is not impacted by replication stress (Fig 1C), this configuration allows for a reasonably accurate estimation of P0 DI values.

### Hydroxyurea treatment

Standard nematode growth media plates containing 10 ml media in a 60 mm petri dish were seeded with 500 ul of an overnight culture of OP50 bacteria. The next day the plates were overlayed with 250 ul of a 100 mg/ml solution of HU (Sigma) and allowed to dry overnight. Plates were used the following day. For treatment, young gravid adults were transferred to the plates and incubated for 3 hours at room temperature prior to analysis.

### UV-C treatment

Young gravid adults were placed in standard nematode growth media plates containing 10 ml media in a 60 mm petri dish. The dishes were then placed on the floor, in the center, of a Stratagene UV Stratalinker 1800 with the covers removed and irradiated at 112 J/m^2^.

## Supporting Information

S1 Fig Worms fed *E*. *coli* containing the RNAi expression vector pL4440 show normal cell cycle timing and normal DNA replication stress responses. Embryos from the indicated strains were optionally treated with either HU or UV-C, as indicated. The time required for embryos to progress from the mid-migration to the NEB stage was then recorded. For each data point, ten embryos were timed over two independent biological replicates (5/replicate) and the values were then averaged and plotted.
(TIF)

S2 Fig Accelerated cell cycles after *wrn-1* RNAi in two-cell embryos. Either N2 or *wrn-1(RNAi)* embryos were timed for interphase duration at the two-cell stage. For each data point, ten embryos were timed over two independent biological replicates (5/replicate) and the values were then averaged and plotted.
(TIF)
