# Supplementary figures and images for: Cell-Type Specific Responses to DNA Replication Stress in Early C. elegans Embryos
Source: PLoS One. 2016 Oct 11;11(10):e0164601. doi: 10.1371/journal.pone.0164601 (PMC5058509; doi:10.1371/journal.pone.0164601)

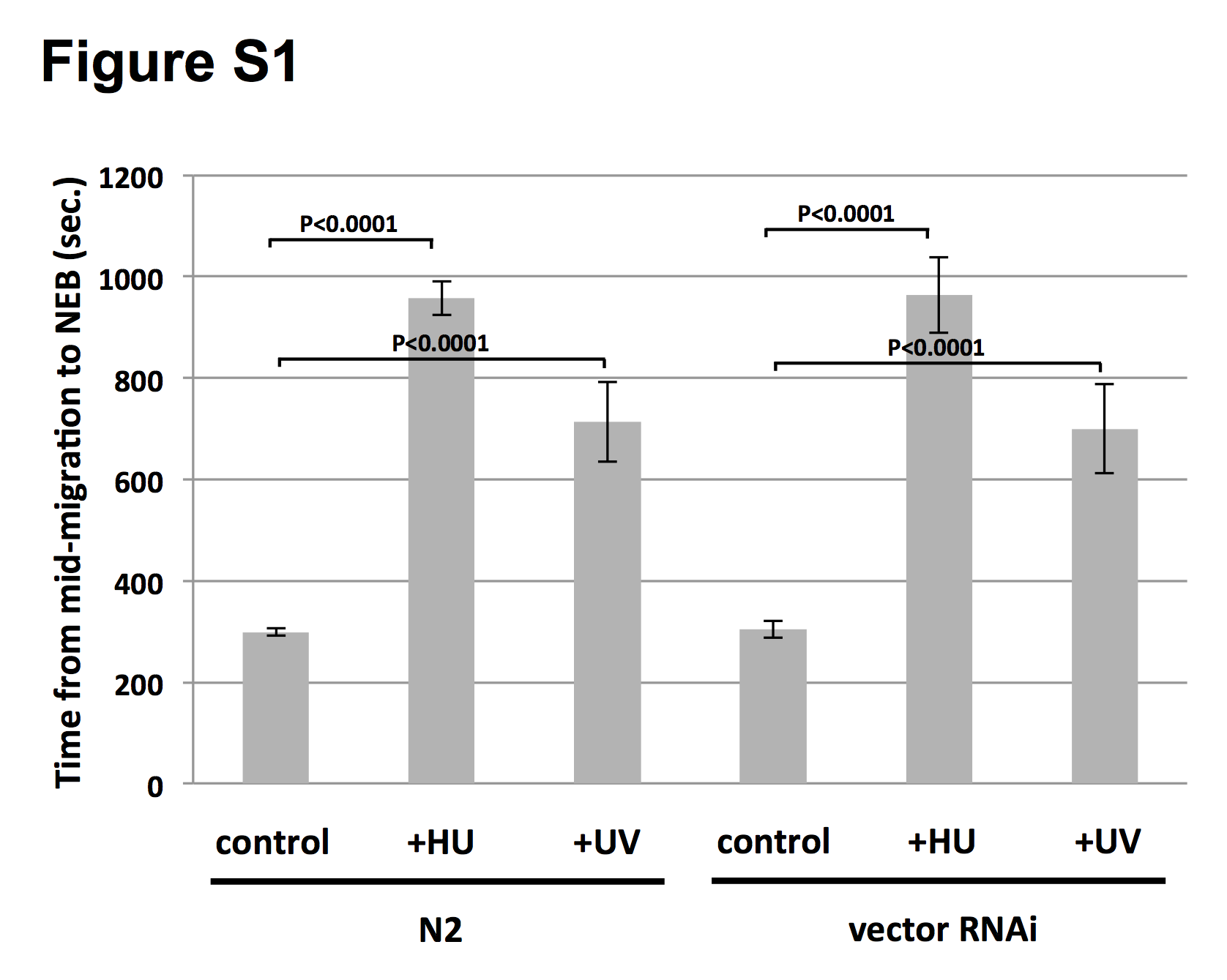

Supplement: S1 Fig — Embryos from the indicated strains were optionally treated with either HU or UV-C, as indicated. The time required for embryos to progress from the mid-migration to the NEB stage was then recorded. For each data point, ten embryos were timed over two independent biological replicates (5/replicate) and the values were then averaged and plotted. (TIF) [file pone.0164601.s001.tif]

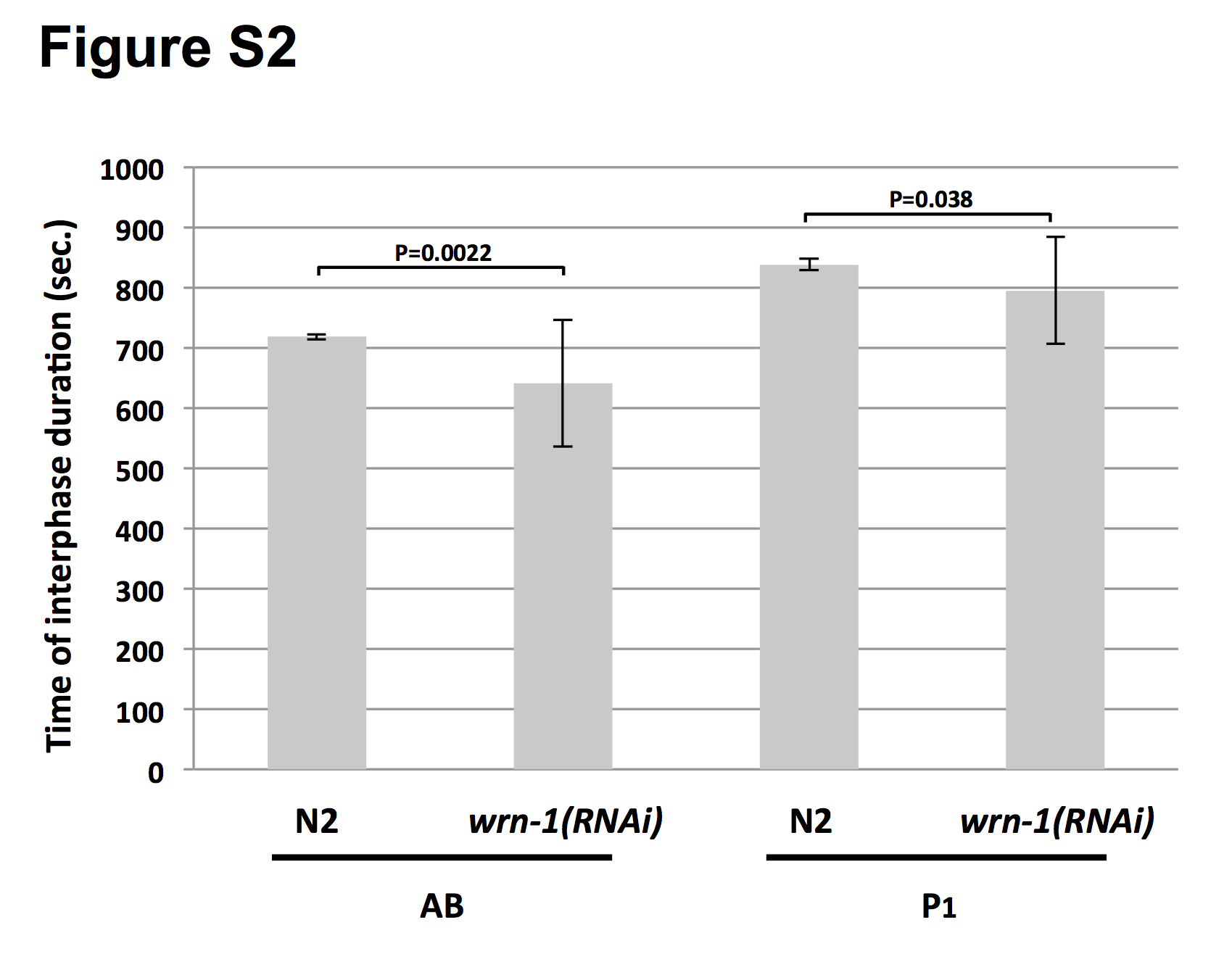

Supplement: S2 Fig — Either N2 or wrn-1(RNAi) embryos were timed for interphase duration at the two-cell stage. For each data point, ten embryos were timed over two independent biological replicates (5/replicate) and the values were then averaged and plotted. (TIF) [file pone.0164601.s002.tif]
